# The neurotrophin Neuritin1 (cpg15) is involved in melanoma migration, attachment independent growth, and vascular mimicry

**DOI:** 10.18632/oncotarget.13585

**Published:** 2016-11-25

**Authors:** Anja Katrin Bosserhoff, Nadja Schneider, Lisa Ellmann, Lucie Heinzerling, Silke Kuphal

**Affiliations:** ^1^ Institute of Biochemistry (Emil-Fischer-Center), Friedrich Alexander University Erlangen-Nürnberg, Erlangen, 91054, Germany; ^2^ Institute for Functional Genomics, University Regensburg, Regensburg, 93053, Germany; ^3^ Institute of Dermatology, University Hospital Erlangen, Friedrich Alexander University Erlangen-Nürnberg, Erlangen, 91054, Germany

**Keywords:** NRN1, melanoma, migration, biomarker, serum

## Abstract

The neurotrophin Neuritin1 (NRN1; cpg15) belongs to the candidate plasticity gene (CPG) family and is expressed in postmitotic-differentiating neurons of the developmental nervous system and neuronal structures associated with plasticity in the brain of human adult.

Our newest findings document that NRN1 deregulation could contribute also to disease development and have impact on malignant melanoma. Our analyses displayed the over-expression of NRN1 in melanoma *in vitro* and *in vivo*, shown by immunohistochemistry and qRT-PCR on microdissected melanoma tissue; furthermore, soluble NRN1 was detectable in tissue culture supernatant and serum of melanoma patients.

To investigate the role of NRN1 in melanoma we performed knockdown, over-expression and recombinant-NRN1-treatment experiments affiliated by functional assays. Our results show that migration, attachment independent growth and vasculogenesis were affected after manipulation of NRN1 on endogenous and extrinsic level. Interestingly, high NRN1 serum levels correlate with low MIA serum levels (< 10ng/ml). Therefore, we speculate that NRN1 could be a marker for early melanoma stages, in particular.

In summary, we detected an overexpression of NRN1 in melanoma patient. In functional cell culture experiments we found a correlation between NRN1 expression and the cancerous behavior of melanoma cells.

## INTRODUCTION

Metastatic melanoma is a very aggressive form of skin cancer and continues to be an intractable disease that usually defies every therapeutic modality if not treated in an early primary state. Melanoma is a major cause of mortality in many developed countries. Advances in overall survival in metastatic melanoma are based on targeted- and immune- therapies.

However, the advances in the knowledge of the pathogenesis of melanoma have allowed the development of new strategies to treat this disease [1]. Along with determining new molecules involved in the pathogenesis of melanoma it is important to examine molecules for their feasibility as melanoma prognostic biomarkers which help to refine the risk of progression and to assess the clinical outcome in immunohistochemistry or serological analysis. A more accurate, therapeutically predictive classification of human melanomas and selection of patient populations that would profit from therapeutic interventions are among the major challenges expected to be addressed in the future. The American Joint Committee on Cancer (AJCC) recommended serum LDH (lactate dehydrogenase) to classify patients with metastatic melanoma in stage IV [2, 3]. However LDH sensitivity and specificity is low. In melanoma elevated serum levels of MIA (Melanoma Inhibitory Activity) and S-100B (beta) are associated with shorter survival and thus were useful as prognostic factors in stages III and IV disease [4–8] and are elevated after recurrence of the disease. Serological analysis are advantageous for the patient therefore it is necessary to consider soluble factors.

One example of such a soluble protein is Neuritin, also termed NRN1 (candidate plasticity-related gene 15, CPG15) which is in focus of our analysis. NRN1 belongs to the family of neurotrophic factors, like nerve growth factor (NGF), brain-derived neurotrophic factor (BDNF), and the neurotrophins NT-3, and NT-4. NRN1 is expressed in postmitotic-differentiating neurons of the developing nervous system and neuronal structures associated with synaptic plasticity in the adult. *In vitro* assays demonstrated that this protein promotes neurite outgrowth and arborisation, dendritic outgrowth, and axonal outgrowth [9–12] suggesting its role in promoting neuritogenesis. In summary, like other neurotrophic factors, NRN1 has multiple roles during nervous system development although, interestingly, it has no sequence homology with traditional neurotrophin ligands.

NRN1 is not only an activity-regulated gene that requires action potential activity for maintenance of synaptic plasticity of the adult brain but it is also expressed in an activity-independent manner during early brain development before circuit formation and maturation, suggesting that it may have different roles. The highly conserved NRN1 protein (142-amino acids long; aa) contains both, a predicted 27-aa secretory signal peptide at its N-terminus and a 27-aa glycosylphosphatidylinositol (GPI) anchor at its C-terminus (from aa 117 to aa 142). Its processed form is attached extracellularly to the cell membrane; thereby it is also defined as membrane-bound ligand. It has also been reported that a soluble form of NRN1 is present in embryonic brain due to phospholipase C “shedding” which promotes the cleavage of the NRN1 GPI anchor and release of GPI-anchored NRN1 from the cell surface. Soluble NRN1 is a paracrine survival factor and protects neurons from apoptosis [13]. Besides its function in the nervous system, NRN1 has also pathogenic and cancerous functions. Two studies implicate NRN1 in emphysema severity and in changes of liver maturation and regeneration [14, 15]. Furthermore, NRN1 is essential for the transformation of endothelial cells by Kaposi's sarcoma-associated herpesvirus [16]. In cancer NRN1 acts as an angiogenic- and hypoxia-induced factor [17, 18] and is associated with proliferation, apoptosis, and angiogenesis of human astrocytoma [19]. In breast cancer the promoter of *cpg15* (*nrn1*) was found to be hyper-methylated [20].

Melanoblasts of the skin and peripheral sensory neurons are two different cell lineages derived from neural crest cells during embryonic development. This common neural crest origin of the nervous system and the melanoblasts of the skin suggest that many molecules and growth factors regulating the development and function of neurons are also involved in the control of skin development. One of these candidates could belong to the neurotrophic factor family. Furthermore, several characteristics of melanoblasts - such as differentiation, proliferation and migration - closely resemble common features of malignant primary and metastatic melanoma cells in many respects. However, in contrast to the strictly controlled mechanisms in embryogenesis, large parts of these common features seem to be unregulated in tumorigenesis. In this context, we found NRN1 to be up-regulated in a cDNA array in melanoblast related cells (MBrc) and melanoma cell lines compared to normal human epidermal melanocytes (NHEM).

Therefore, in this study we analyze the role of NRN1 in the biology of melanoma. We show that NRN1 is nearly not expressed in normal human melanocytes (NHEM) but melanoma cells showed an elevated NRN1 expression level. Silencing of NRN1 in melanoma cells led to reduced migration and attachment independent growth in 3-D soft agar assay. Interestingly, we found also chemotactic influences of soluble NRN1, as the induction of vascular structures by melanoma cells in angiogenesis assays. The results suggest that NRN1 expression may be part of oncogenic signaling pathways in melanoma.

## RESULTS

### Expression of NRN1 in melanoma cell lines

**Figure 1:**
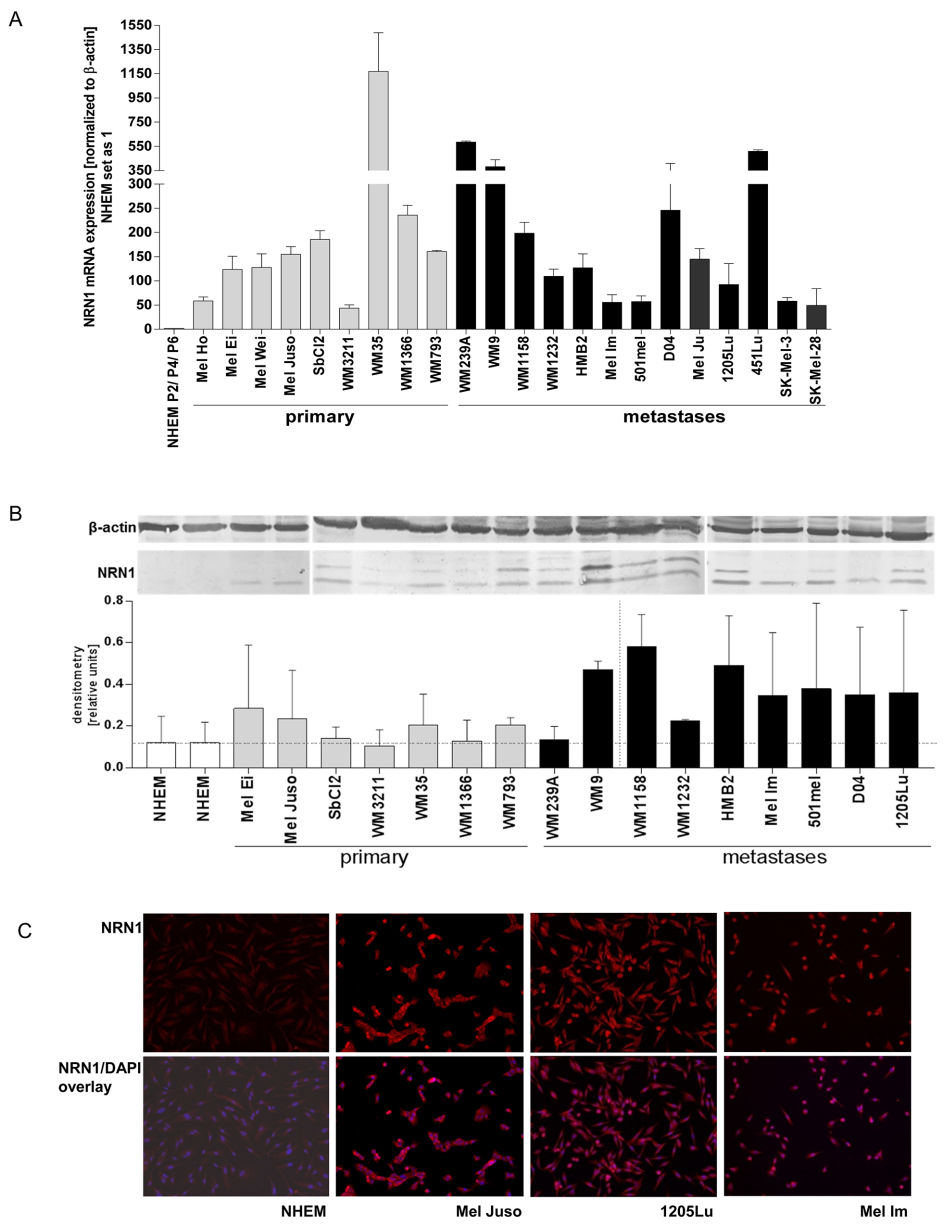
NRN1 expression in melanoma cell lines **A.** Quantitative real time PCR analysis for NRN1 (*cpg15*) expression in melanoma cell lines (grey bars, 9 primary melanomas; black bars, 13 melanoma metastases) in comparison to three different donors of normal human epidermal melanocytes isolated from human foreskin (NHEM) which were set as one. Data is mean +/− SE from triplicate experiments. **B.** Representative western blot analysis of different melanoma cell lines which show the NRN1 expression (mature and precursor protein) in comparison to NHEM. β-actin was used as loading control. The densitometry was calculated for combining the two bands of NRN1. **C.** Immunofluorescence staining for NRN1 (red) in three melanoma cell lines and NHEM. The overlay images combine DAPI staining with NRN1 staining.

To evaluate whether malignant transformation affects the expression of NRN1, we analyzed NRN1 in melanoma cells both at the mRNA and at the protein level. Melanoma cells synthesize NRN1 in higher amounts than melanocytes from different donors and in different passages (NHEM P2/P4/P6), as shown by qRT-PCR on nine primary and thirteen metastatic melanoma cell lines (Figure 1A), western blot analysis of protein lysates of sixteen melanoma cell lines (Figure 1B), and immunofluorescence staining of three melanoma cell lines (Figure 1C). Differences between mRNA and protein levels may be explained by the discrepancies between regulation of mRNA and protein stability however, this was not in focus of this study.

The two specific bands observed in the western blots reflect the expression of the mature and the processed (without GPI anchor) NRN1. Strikingly, mainly the processed form of NRN1 is observed in Mel Ei, Mel Juso, Mel Im, and D04. The densitometry (Figure 1B) is normalized to β-actin and combines both bands. In addition, we analyzed neurotrophins NT-3, NT-4, brain-derived neurotrophic factor (BDNF), and nerve growth factor (NGF) on mRNA level in seven melanoma cell lines compared to NHEM (supplementary Figure S1A). Melanoma cells express all the other neurotrophins to a nearly similar expression level like NHEM. Furthermore, a cDNA array comparing three primary melanomas and three melanoma metastases with NHEM showed that NGF, NT-3, NT-4, BDNF, and NRN1-like are not differentially regulated and only NRN1 is ~48-fold elevated in melanoma compared to NHEM (supplementary Figure S1B).

### Regulation of NRN1 by hypoxia

**Figure 2:**
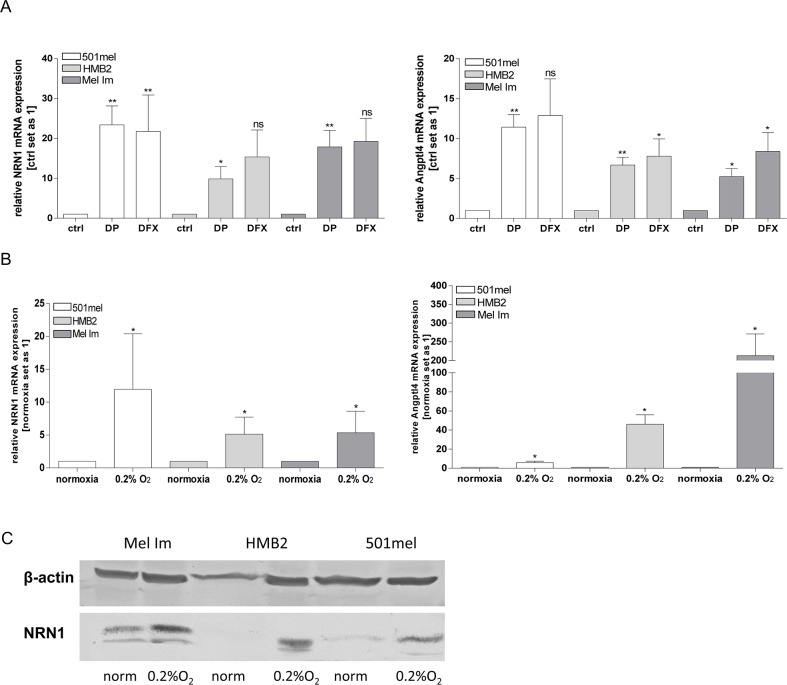
Hypoxia-induced expression of NRN1 **A.** mRNA expression of NRN1 and of the positive control for hypoxia: Angptl4; after treatment of melanoma cell lines with solvent (ctrl), 2,2′-dipyridyl (DP) and Desferrioxamine (DFX). (Statistical analyzes by unpaired t-test, *p:<0.05; **:p<0.01; ns, not significant). **B.** and **C.** For comparison cells were also exposed to normoxia and hypoxia (0.2% O_2_) and the mRNA and protein status of NRN1 was analysed. (Statistical analyzes by unpaired t-test, *p:<0.05; **:p<0.01; ***:p<0.001).

In the literature it was demonstrated that NRN1 can be regulated by hypoxia [17], a condition which is cumulative and endogenously found in melanomas. A previous publication of our own group revealed constitutively induction of HIF-1α expression in melanoma cells [21]. Therefore, we investigated the hypoxic effects on NRN1 expression using Desferrioxamine (DFX) and 2, 2′-dipyridyl (DP) as iron chelators and inhibitors of prolyl hydroxylases (PHDs). Both chemical compound simulate hypoxic effects and increase the NRN1 amount on mRNA level as exemplarily shown in the melanoma cell lines 501mel, HMB2, and Mel Im (Figure 2A). Stimulating effects of DP were significantly inducing NRN1 expression whereas DFX was not as effective as DP. Under real hypoxic conditions of 0.2 % oxygen and 5 % carbon dioxide for 48 h the melanoma cell lines showed elevated expression level of NRN1 on mRNA and protein level which confirmed the previous results (Figure 2B and 2C). The molecule Angptl4 serves, as described in the literature, as positive control for hypoxic conditions (Figure 2A and 2B).

### NRN1 modulate the oncogenic behavior of melanoma cells

To analyze whether NRN1 actually exerts any function on melanoma cells, we decided to focus our attention on three melanoma cell lines: HMB2, 501mel, and Mel Im which are well known in the melanoma research field and show high expression levels of NRN1. To assess the role of NRN1 on cell proliferation, we performed siRNA silencing experiments using two different siRNAs (_6, _7) against NRN1 in transient transfection (48 h) experiments. The successful knockdown on mRNA and protein level in the three melanoma cell lines are shown as Supplementary Figure S2A and S2B. Cell growth was measured in an *in vitro* real-time proliferation assay using the xCELLigence system and the results of three independent experiments were summarized in Supplementary Figure S2C. Interestingly, proliferation was unaffected by NRN1 modulation as well as short time (~30 min) attachment behavior (supplementary Figure S2D).

**Figure 3:**
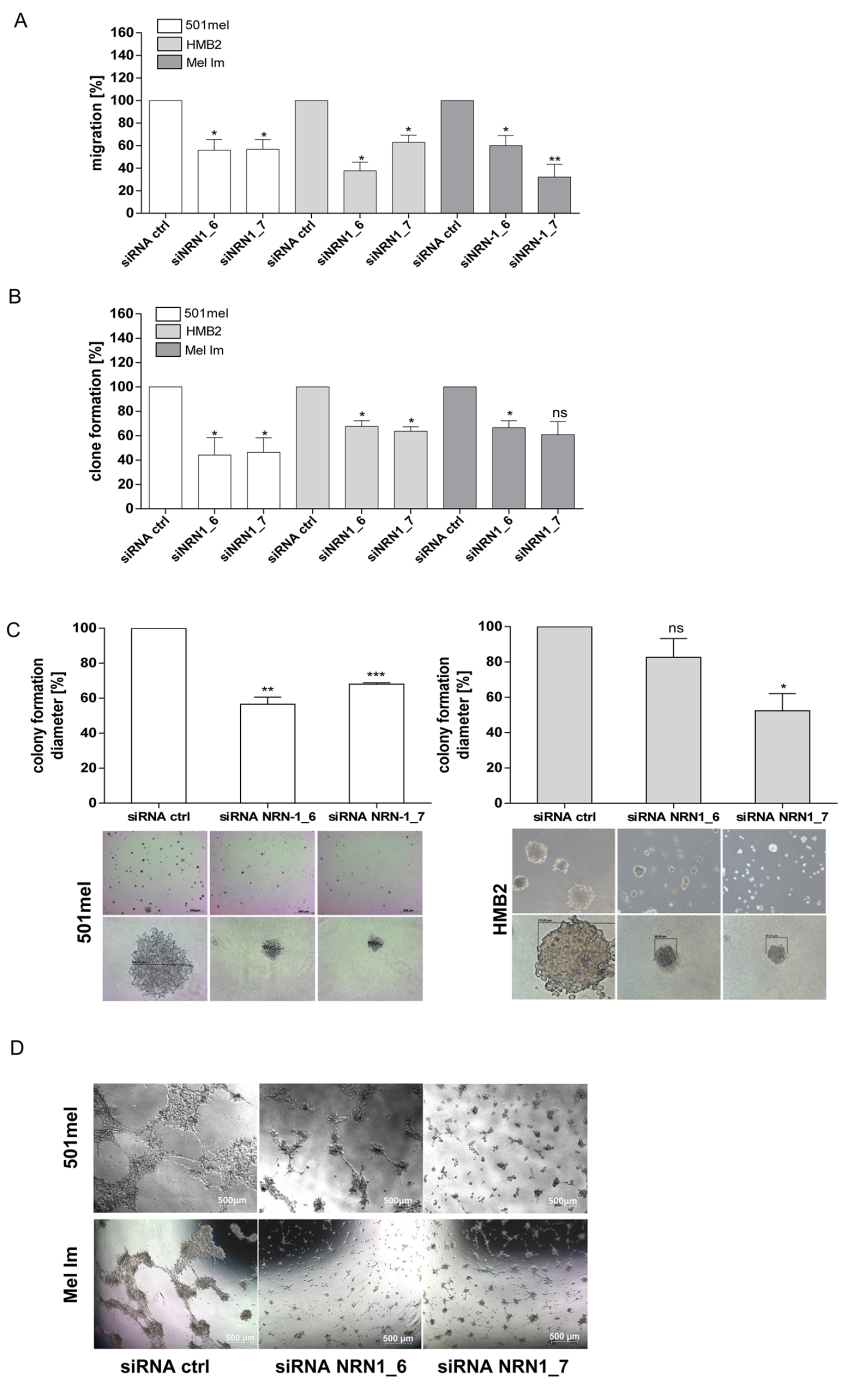
Functional analysis of the transient knockdown of NRN1 **A.** xCELLigence system for analyzing the migration of 501mel, HMB2, and Mel Im cells after knockdown of NRN1 (siNRN1_6, siNRN1_7) compared to control siRNA transfected cells (siRNActrl). In the lower chamber fibroblast conditioned medium (FCM) was used as chemoattractant. (*p:<0.05; **:p<0.01). **B.** Clonogenic assay after knockdown of NRN1 (siNRN1_6, siNRN1_7) compared to control siRNA transfected cells (siRNActrl). (*p:<0.05; **:p<0.01***; :p<0.001; ns, not significant). **C.** and **D.** 3-D assays: colony- and tube formation after knockdown of NRN1 (siNRN1_6, siNRN1_7) in two different melanoma cell lines (501mel, HMB2) compared to control siRNA transfected cells (siRNActrl). (*p:<0.05; **:p<0.01; ***:p<0.001; ns, not significant).

Melanoma is notorious for its high tendency to metastasize by migration. Next, we therefore measured the migration capacity with the xCELLigence system after silencing NRN1 expression. Loss of NRN1 decreases the migration rate in the cell lines to approximately 50% (Figure 3A). Additionally, we used the clonogenic assay as the method of choice to test the survival rate based on the ability of a single cell to grow into a colony. The assay essentially tests every cell in the population for its ability to undergo “unlimited” division. SiRNA against NRN1 effectuate the stem cell behavior and clone formation capacity of 501mel, HMB2, and Mel Im cells in a negative manner (Figure 3B). Anchorage-independent growth, the cell's ability to proliferate without attachment to or spreading onto, a substratum, is one of the hallmarks of transformation and the most accurate *in vitro* indication of tumorigenicity. For this 3-D assay we used two melanoma cell lines, as these were best suited for this assay. Using this soft agar assay we could show that NRN1 down-regulation prevents colony formation up to ~50 % (Figure 3C) in 501mel and HMB2. In a further 3-D assay of tube formation we defined the influence of NRN1 on vascular mimicry of melanoma cells. As HMB2 does not form tubes in this assay it was necessary to replace HMB2 by the cell line Mel Im. In the two mentioned cell lines NRN1 knockdown led to reduced tube formation on Matrigel (Figure 3D).

### Recombinant soluble NRN1 and its functions in melanoma

**Figure 4:**
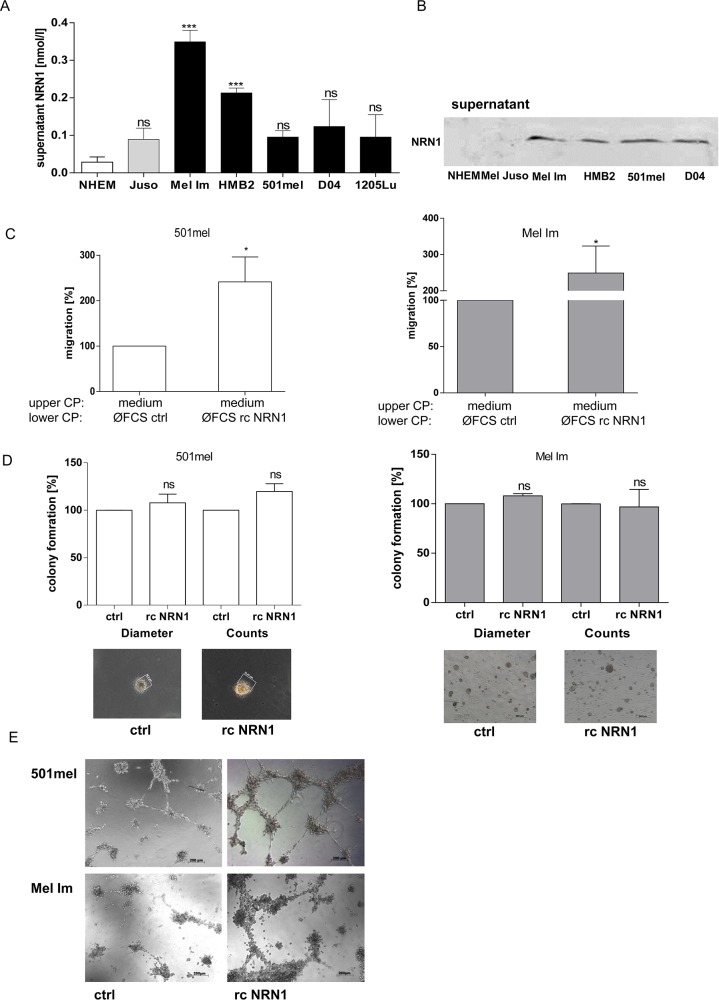
Soluble NRN1 **A.** ELISA measurement of supernatant of different melanoma cell lines (primary melanoma, grey bar; melanoma metastases, black bars) and NHEM (white bar). (Statistical analyzes by unpaired t-test, ***;:p<0.001; ns, not significant). **B.** Western blot analysis of supernatant of NHEM and four melanoma cell lines. **C.** xCELLigence system for analyzing the migration of 501mel and Mel Im cells using recombinant (rc) NRN1 in the lower compartment of a well chamber system. (*p:<0.05). **D.** and **E.** 3-D assays: colony- and tube formation after treatment of 501mel and Mel Im with recombinant (rc) NRN1 diluted in DMEM medium.

As previously mentioned NRN1 also exists as soluble form in conditioned medium of neuronal cells *in vitro* as well as in the extracellular matrix of neurons *in vivo*. Therefore, we used first an ELISA to quantify NRN1 in the supernatant of different melanoma cells and detected elevated NRN1 levels compared to NHEM (Figure 4A). Further, we validated the occurrence of soluble NRN1 by western blot analysis of supernatants of NHEM and melanoma cell lines and confirmed the ELISA result (Figure 4B). Melanoma cell migration and invasion are controlled in various stages of tumor progression by different soluble factors (e.g. growth factors and cytokines), which by autocrine and paracrine effects enable cells to grow autonomously and confer competence to metastasis [22]. Therefore, we used recombinant NRN1 to prove its effectiveness to regulate tumor-promoting effects on melanoma cells. Recombinant (rc) NRN1 does not regulate endogenous NRN1 expression level (Supplementary Figure S3A) or the proliferation rate and short time (~30 min) attachment (Supplementary Figure S3B and S3C) of melanoma cells. To asses NRN1 as chemoattractant we used again the xCELLigence system for migration assays. Here, the melanoma cells in the assay were kept in fresh DMEM medium, hence the medium was not already enriched with (cellular)-secreted NRN1. In this analysis rcNRN1 in the lower chamber of a well system significantly attractated 501mel and Mel Im cells to migrate (Figure 4C). Changes of the stem cell-like character of melanoma cells were not detectable in the clonogenic assay after treatment of the cells with rcNRN1 (data not shown). In two different 3-D assays, namely colony formation in soft agar and tube formation, we further analyzed the oncogenic character of soluble NRN1. In contrast to the siRNA experiments, rcNRN1 has no influence on attachment independent growth in the soft agar assay (Figure 4D). Potentially, the recombinant protein cannot be used in agar and in addition, in this long term assay the high abundance of soluble NRN1 prevents significant effects of rcNRN1.

Nevertheless, the investigation of the angiogenic activity of NRN1 on melanoma cells showed that rcNRN1 drastically induces vasculogenic mimicry of melanoma cells (Figure 4E).

### Chemotactic effects of NRN1 on human primary melanocytes

**Figure 5:**
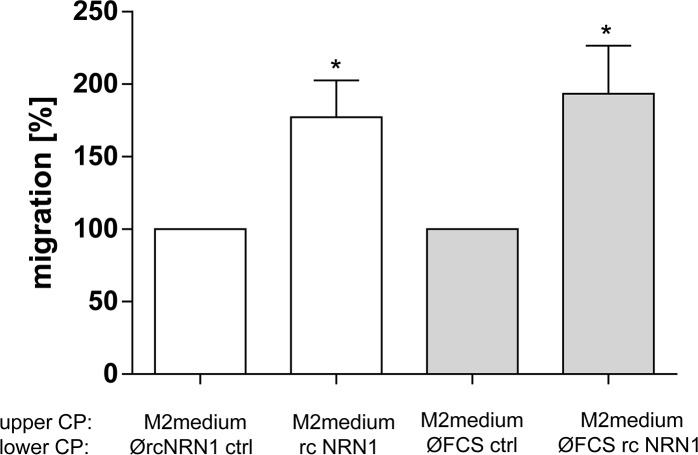
NRN1 in melanocytes xCELLigence system for analyzing the migration of primary NHEM using recombinant (rc) NRN1 in the lower compartment of a well chamber system diluted with M2 medium and DMEM medium without FCS, respectively (lower compartment (lower CP). (*p:<0.05). NHEM were counted and cultivated in M2 medium (upper compartment, upper CP).

The previous experiments revealed the tumor-promoting influence of rcNRN1 on melanoma cells. Therefore, we were also interested to assess the reaction of primary normal human epidermal melanocytes (NHEM) to rcNRN1 as attractant, as these cells express nearly no NRN1. We performed xCELLigence experiments to analyze the migration of NHEM and demonstrated their migration into the direction of the attractive gradient of NRN1 (Figure 5).

### Stable over-expression of NRN1 in melanoma

After sub-cloning NRN1 into the eukaryotic expression vector pCDNA3 we stably transfected the melanoma cell line Mel Ju and proved the over-expression on mRNA and protein level in two different cell clones (NRN1_a and NRN1_b) compared to two controls (ctrl a and ctrl b) (Supplementary Figure S4A and S4B). In functional assays short time attachment and proliferation were unaffected by NRN1 over-expression (Supplementary Figure S4C and S4D). The migration rate was also not significantly influenced by NRN1 over-expression. The NRN1 cell clones _a and _b showed only a slight tendency for an increased migration rate (Supplementary Figure S4E). In 3-D assays (tube formation, colony formation) the mentioned cell clones did not influence the vessel or colony density (data not shown).

### NRN1 *in situ* and *in vivo*

**Figure 6:**
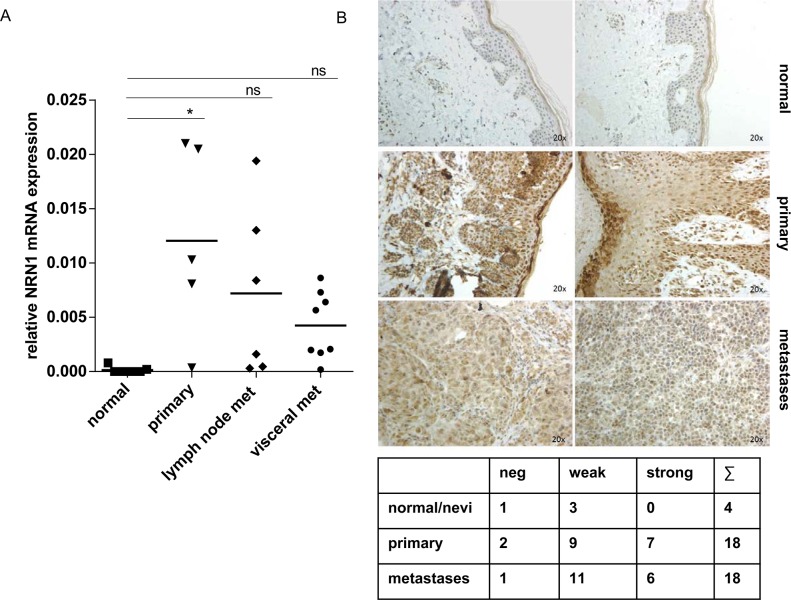
*In vivo* relevance of intrinsic NRN1 **A.** Quantitative real time PCR of micro-dissected melanoma tissues from different patients. Melanocytes and normal skin are in comparison to 5 primary melanoma tissues and 14 melanoma metastases (lymph node and visceral). Data is mean +/− from triplicate experiments. (*p:<0.05). **B.** Immunohistochemistry of a tissue microarray (TMA) with positive NRN1 staining in melanoma samples (primary and metastases). The table gives an overview of the immunohistochemistry of the whole TMA.

To extend our experiments, we further analyzed NRN1 mRNA expression level from *in situ* microdissected tissue from melanoma patients (5 primary, 6 lymph node- and 9 visceral metastases) and compared the expression status with NHEMs and microdissected epidermis from normal skin (altogether 7 donors) (Figure 6a). The *in situ* data also showed the high expression of NRN1 in primary tumors and metastases. Particularly, primary tumors showed a significantly higher mRNA expression level compared to lymph node- and visceral metastases (Figure 6A). The immunohistochemical staining of a tissue microarray revealed the protein occurrence of NRN1 particularly in primary melanoma and metastases, whereas the overall impression of a stronger staining for NRN1 in primary tumors was observed (Figure 6B). In general, weak to strong staining of NRN1 were visible in sixteen of eighteen primaries and seventeen of eighteen metastases. Keratinocytes of the epidermal layer were also positive for NRN1 (normal skin, and primary melanoma). We also aimed at detecting the NRN1 double band in protein lysates of melanoma tissue (Supplementary Figure S4F). *In vivo* three of six samples showed the double band of NRN1 in western blot analysis, one melanoma sample showed the single band of processed NRN1. Two melanoma samples were negative for NRN1 and two normal skin protein lysates (N) were also negative for NRN1.

**Figure 7:**
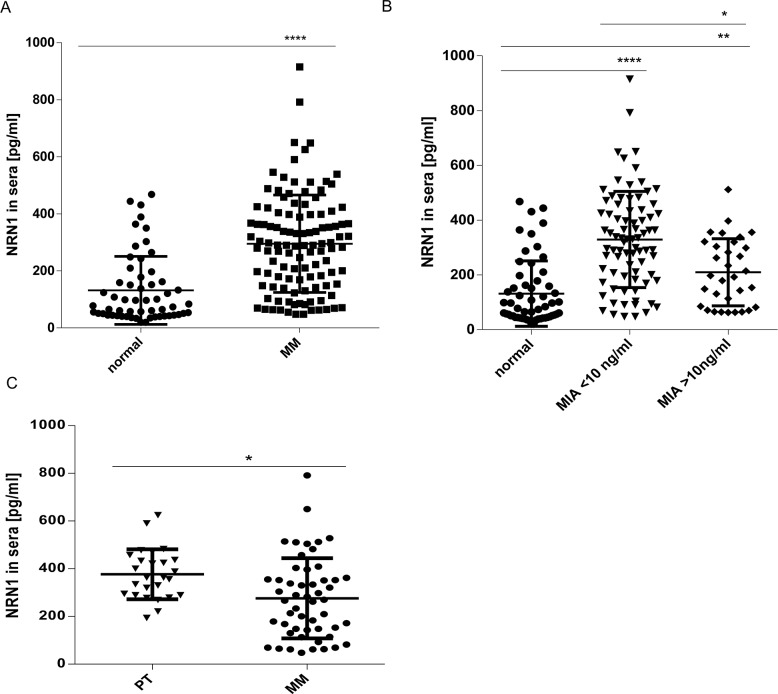
*In vivo* relevance of secreted NRN1 **A.** ELISA measurement of NRN1 in 170 sera of healthy (57) - and cancer (113) suffering donors. (MM, malignant melanoma; ****:p<0.0001). **B.** Clustering of NRN1 ELISA results in sera of cancer suffering donors compared to the ELISA-measured MIA status. (*p:<0.05; **:p<0.01; ****:p<0.0001). **C.** Clustering of NRN1 ELISA results in sera of cancer suffering donors with primary melanoma and melanoma metastases. (*p:<0.05).

Due to the fact that the experimental procedures for *in vivo* analyses were focused on intrinsic NRN1 we subsequently performed ELISA technology to measure secreted NRN1. Here, sera of 113 melanoma patients in melanoma stage I-IV were compared to 57 healthy donors (Figure 7A). Interestingly, the baseline NRN1 values in healthy donors ranged from ~20 to ~390 pg/ml and NRN1 was significantly elevated in the melanoma patient sera (max 915 pg/ml) compared to the healthy individuals. For the melanoma patient sera we classified the NRN1 values corresponding to the melanoma marker MIA (the relevance was mentioned in the introduction) and found a significant correlation to MIA serum levels < 10 ng/ml (Figure 7B). 78 from 113 melanoma patients were classifiable in the categories primary melanoma and metastases (Figure 7C). The sub-division showed the result that patients with primary melanoma have more NRN1 in their serum compared to patients with melanoma metastases.

Unfortunately, because of ethical guidelines it was not possible to get survival information from melanoma patients considering NRN1. Therefore, we used TCGA datasets considering alternative cancer entities. Analysis of these TCGA datasets (using PRISURV) [23, 24] revealed longer survival of breast cancer, lung cancer, and cervical cancer patients with high as compared to low NRN1 expression (Supplementary Figure S5A). We chose to include all cancer GEO datasets with a P-value lower than 0.055.

## DISCUSSION

In this study we show that melanoma patients have significantly higher NRN1 level compared to non-melanoma patients.

Most of the previous manuscripts about neurotrophins deal with the receptors TrkA, TrkB, TrkC, and p75NTR (CD271) in healthy and diseased skin [25, 26] and consider the ligands to a lesser extent. A well-known example is the analysis of p75NTR (CD271), also known as NGF receptor (NGFR), which has been shown to be specifically expressed on neural crest cells from which melanocytes derive. Of note, several reports have shown that the CD271 positive melanoma cells harbor melanoma initiating capacities (a stem cell like character) with a higher tumorigenic potential when injected into nude mice [27, 28]. The so far known further receptors for neurotrophins (TrkA, TrkB, TrkC) were also expressed by several analyzed melanoma cell lines [29, 30].

Furthermore, neurotrophins were discussed to augment the production of heparanase, an important and unique extracellular matrix degradative enzyme in brain metastasis of melanoma cells [31–33]. In agreement with further publications [29, 30] we show by qRT-PCR that neurotrophins NGF (nerve growth factor), NT-3, NT-4 (neurotrophins 3 and 4), and BDNF (brain-derived neurotrophic factor) are not only derived from other skin-cells like fibroblasts and keratinocytes, but are produced to different extends by melanoma cells itself (Supplementary Figure S1).

In particular, the expression of a newer member of the neurotrophin family, NRN1 (cpg15), is differentially regulated between NHEM and melanoma cell lines which is shown for the first time by our analysis on mRNA and protein level. Here, the double band observed in the western blots reflects the expression of the mature and the processed NRN1 whereat we only speculate that the processing could be different in each cell line.

Importantly, the increased *in vivo* expression of internal NRN1 in primary melanoma and metastases versus normal or nevi tissue was also detectable by our research of laser-micro-dissected melanomas of patient and by immunohistochemistry analysis of melanoma samples (Figure 6).

The expression of NRN1 was originally connected to survival, differentiation, function and repair of neurons. Up to now, only three manuscripts found a relationship between NRN1 and cancer where it was associated with angiogenesis, proliferation, and apoptosis of astrocytoma, Kaposi's Sarcoma, and cancer angiogenesis in general [16, 18, 19]. The manuscripts also revealed a guidance function of a soluble and secreted form of NRN1 that could be of importance for cancer cells. Therefore, we analyzed the occurrence of soluble NRN1 in the supernatant of melanoma cells. An ELISA and Western blot analysis detect secreted NRN1 in several melanoma cell lines. Whether this melanoma relevant and interesting member of the neurotrophin family signals also via the known and above mentioned receptors is unknown, so far.

Our further analyses considered the function of soluble and intracellular NRN1 in experiments with recombinant protein, siRNA knockdown, and over-expression trials. Surprisingly, neither attachment nor proliferation are influenced by recombinant NRN1 and after knockdown of NRN1, respectively, which is in contrast to the results made in astrocytoma [19]. Accompanied with the data of Raggo et al. [16] anchorage-independent growth in a 3-D assay is regulated by recombinant NRN1 and after knockdown of NRN1 in our analyzed melanoma cells. The migratory- and angiogenic capacity of melanoma cells was strikingly regulated by recombinant NRN1 and after silencing of endogenous NRN1, respectively. Both cellular properties are also conducive for vasculogenic mimicry of melanoma. Our data build on previous results where NRN1 act as guidance factor for neurons and endothelial cells whereat also melanoma cells were directly guided by NRN1 to form endothelial like structures. As also recombinant NRN1 has the above mentioned functional capacity it seems that melanoma cells harbor the expression of a receptor of the ligand NRN1. Whether CD271 could be the specific receptor for NRN1 or if it's one of the other receptors (TrkA, TrkB, TrkC) or a so far not detected one, is unknown. The cellular effect of neurotrophins depend on the expression level of the neurotrophin and also the binding affinity to the particular receptor and makes the search for a specific receptor for NRN1 very complex and was not in focus of this manuscript.

Furthermore, also NHEM respond to recombinant NRN1 in migration assays with a higher migration rate after using NRN1 as chemoattractant. Whether a known neurotrophin receptor is responsible for binding the ligand NRN1 is not known through publications by other groups and was not analyzed by us. It was only previously described that melanocytes express p75NRT, TrkA, and TrkC [26, 34].

It was previously mentioned that NRN1 could be regulated by hypoxia in human micro-endothelial cells (HMEC-1) [17]. Therefore we incubated melanoma cells with medium enriched with Desferrioxamine (DFX) and 2, 2′-dipyridyl (DP), respectively to simulate hypoxic conditions. Furthermore, we compared the expression level of NRN1 under low oxygen conditions with normal oxygen conditions. Both treatment strategies for melanoma cells led to elevated NRN1 expression levels. Whether the NRN1 expression level is also regulated by miR-204 in melanoma was not further analyzed by us. However, Gao and colleagues described the suppression of NRN1 via miR-204 in rat Schwann cells [35]. This is in agreement with the detection of low expression levels of miR-204 in melanoma [36] eventually accompanied by high NRN1 levels. The deduction is that low miR-204 levels in melanoma allow a high expression rate of NRN1.

Extracellular tumor markers may have potential role in the follow-up of patients with malignant melanoma, in therapy monitoring and in prediction of prognosis and would increase the accurateness of staging in these patients. It is desirable to have a diagnostic marker at least to follow the progression of the disease. Therefore, we started to analyze serum-NRN1 in an ELISA system to proof its tumor marker capability. We defined the NRN1 secretion in 113 melanoma and 57 normal patient sera. The melanoma patients harbor significant higher NRN1 levels compared to non-melanoma patient (Figure 7A). For the different melanoma patient sera we classified the NRN1 values corresponding to the melanoma marker MIA. As result we found a significant correlation to MIA serum levels <10 ng/ml (most laboratories use a cut-off level of MIA of 6.5ng/ml). Furthermore, the categorization of secreted NRN1 in patient sera led to the assumption that NRN1 serum levels are obviously rather associated with early tumor stage than late stages. We conclude that it is worth to pursue NRN1 as early tumor marker as the diagnostic procedure of determining the NRN1 serum level is inexpensive and readily performed. For the future is interesting to perform parallel determination of several serum markers, such as MIA, S100-B, and LDH and NRN1 to increase the prognostic value of the analysis.

Unfortunately, cancer survival data from melanoma patients to consider the role of NRN1 in our experimental set were not available to us. However, we used PRISURV as bioinformatics tool uncovering the role of NRN1 in different cancer entities and found a positive correlation of high NRN1 expression levels with cancer survival outcome of breast cancer, lung cancer, and cervical cancer patients supporting our data.

In summary, NRN1 is expressed more extensively in melanoma than in normal melanocytes and healthy tissue. Secreted NRN1 seems to play a role also in earlier phases of melanoma development as we can discover NRN1 over-expression to be associated to primary melanoma (Figure 7B and 7C). Eventually, secreted NRN1 is usable as serum marker for early melanoma. Here, in which further detailed studies are necessary.

## MATERIALS AND METHODS

### Cell culture

The human melanoma cell lines 501mel, HMB2 and Mel Im were derived from metastases of malignant melanomas. Cells were maintained in DMEM supplemented with penicillin (400 U/ml), streptomycin (50 μg/ml), L-glutamine (300 μg/ml) and 10 % fetal calf serum (FCS) and split at a 1:5 ratio every three days. The cells were cultured under a humidified atmosphere of 8 % CO_2_ at 37°C. Further human melanoma cell lines were a kind gift by Meenhard Herlyn (Wistar Institute, Philadelphia, PA) and maintained in a culture medium consisting of MCDB153 (Sigma Aldrich) with 20 % Leibovitz's L-15 (PAA Laboratories, Coelbe, Germany), 2 % FCS (PAA Laboratories), 1.68 mM CaCl_2_ (Sigma Aldrich) and 5mg/ml insulin (Sigma Aldrich). Tissue origins of metastatic melanoma cell lines were the lymph nodes (WM239a, WM9, WM1158, and WM1232) and lungs (451Lu and 1205Lu). Primary melanocytes (NHEM) were isolated from human foreskins and purchased from PromoCell (Heidelberg, Germany) and cultured in Growth Medium M2 (C-24300).

Recombinant NRN1 was purchased from Sigma-Aldrich. Human NRN1 was expressed in *E.coli* and was diluted in PBS buffer containing 0.1 % BSA as a carrier protein as was used in a concentration of 50 ng/ml.

### Analysis of mRNA expression by quantitative PCR

Total cellular RNA was isolated from cultured cells using the RNeasy kit (Qiagen). cDNAs were generated by reverse transcriptase reaction performed in 20 μl reaction volume containing 500 ng of total cellular RNA and p(dN)6 Random Primer (Roche Division Diagnostics, Mannheim, Germany). qRT-PCR was performed on a Lightcycler480 (Roche). cDNA template (500 ng), 0.5 μl (20 mM) of forward and reverse primers and 2 μl of SybrGreen Master I LightCycler Mix in a total of 20 μl were applied to the following PCR program: 30 sec 95°C (initial denaturation); 20°C/sec temperature transition rate up to 95°C for 15 sec, 3 sec 60°C, 5 sec 72°C, 81°C acquisition mode single, repeated for 40 times (amplification). The specific PCR amplification reaction was evaluated by melting curve analyzed and checking the PCR products on 1.8 % agarose gels. Oligonucleotide primers used in the PCR were as follows: NRN1 for: 5′-GGGCGACAGCATGGCCAACT- 3′ and NRN1 rev: 5′- CCGCTGCCGCAGAGTTCGAA- 3′; actin for: 5′-CTACGTCGCCCTGGACTTCGAGC- 3′ and actin rev: 5′- GATGGAGCCGCCGATCCACACGG- 3′. Angptl4 for: 5′-CAGGGTACCTAAGAGGATGAGCGGTGC and Angptl4 rev: 5′-CTGCTCGAGCTGCAGGAGTCCGTG C - 3′. Relative gene expression was expressed as a ratio of the expression level of the gene of interest to that of β-actin.

### Transfection experiments

Cells were plated 2 × 10^5^ cells/well into 6-well plates and transfected with 0.5 μg plasmid DNA using the lipofectamine plus method (Invitrogen, Darmstadt, Germany) according to the manufacturer's instructions. The NRN1 expression construct was received from the group of Elly Nedivi (Cambridge, USA). pFUI**C**GW lentiviral vector with IRES-EGFP-CPG15-FLAG sequence leads to over-expression of NRN1 (CPG-15). We sub-cloned a 580 nucleotide sequence of the NRN1 core and FLAG sequence (EcoRI restriction) into the vector pCDNA3 to stably express NRN1 in our melanoma cell lines. The positive cell clones are under G418 (Sigma Aldrich, Steinheim, Germany) selection (20μg/ml).

Small interfering RNA (siRNA)-mediated down-regulation of NRN1 was achieved by siRNA from Qiagen (Hs_NRN1_6 and Hs_NRN1_7, Qiagen, Hilden, Germany) and “AllStars negative control siRNA” was used for control (Qiagen).

### Western blotting

Cells were lysed in 200 μl RIPA-buffer (50 mM Tris/HCl pH 7.5, 150 mM NaCl, 1% N-P40, 0.1 % SDS, 0.5 % sodium deoxycholate and protease inhibitor) and incubated for 15 min at 4°C. Insoluble fragments were removed by centrifugation at 13′000 rpm for 10 min and supernatant lysates were immediately shock-frozen and stored at −80°C. Lysates (40 μg protein per lane) were resolved on 10 % SDS-PAGE gels and blotted onto PVDF membranes. Membrane blocking was achieved by incubation with 3 % BSA/TBS/0.1 % Tween for 1 h. The following primary antibodies were used: monoclonal anti-NRN1 (1 in 1000 dilution; Novus Biologicals, Abingdon, UK, distributed by R&D Systems, Wiesbaden, Germany), and anti-β-actin (1 in 5000 dilution, Sigma Aldrich). Protein levels of β-actin were analyzed as a control for constant loading and transfer. Incubation with primary antibodies was for 16 h. After three washes with TBS, membranes were incubated for 1 h with an alkaline phosphatase-coupled secondary anti-mouse in TBS/0.1 % Tween (1 in 3000 dilution, NEB, Frankfurt a.M., Germany). Immunoreactive proteins were visualized by NBT/BCIP (Sigma Aldrich) staining.

### Fluorescence

5 × 10^5^ cells were grown on eight-well chamber slide for one day, washed with PBS and fixed with 4 % paraformaldehyde for 15 min. For permeabilisation of the cell membrane, the cells were incubated for 5 min with 0.1 % Triton-X-100 (Sigma Aldrich), washed again and covered with blocking solution (1 % BSA/PBS) for 1 h. Thereafter, cells were incubated with a 1:20 dilution of anti-NRN1 antibody for 1 h at 37°C. After washing the cells, they were incubated with a 1:40 dilution of secondary antibody (FITC anti-mouse, Dako, Hamburg, Germany) in PBS for 1 h followed by washing again. After mounting with Hard Set Mounting Medium with DAPI-Vectashield, H-1500 (Vector Laboratories, Burlingame, USA) images were collected by fluorescence microscopy.

### Immunohistochemistry of melanoma tissue microarray

Formalin fixed, paraffin embedded human melanoma biopsies were obtained from the Institute of Pathology, University Hospital Regensburg, Germany. The medical ethical committee of the University of Regensburg approved the usage of the tissue, and as prescribed patient identity and data privacy was protected. A tissue microarray (TMA) was constructed as described before [37]. Following deparaffinizing in xylene, and rehydration in a graded series of isopropanol, antigen retrieval was achieved by microwave in Tris-EDTA buffer for 5 min. After a peroxidase block (Dako, Hamburg, Germany) sections (5 μm) were incubated with primary monoclonal anti-NRN1 antibody (Novus Biologicals distributed by R&D Systems; 1 in 20 dilution) for 30 min at room temperature. Slides were then washed three times with PBS before incubation with HRP labelled polymer which is conjugated with anti-mouse secondary antibody (Dako EnVision), before again washing three times with PBS. Staining was with DAB (Dako) for 5 min followed by counterstaining with hematoxylin (Merck, Darmstadt Germany).

For isolation of micro-dissected tissue the skin biopsies were also collected in the Molecular Pathology of the University Hospital Regensburg under the above mentioned ethical conditions. Tissue samples from primary human melanoma, and melanoma metastases obtained from patients undergoing surgical treatment, were immediately snap frozen and stored at −80°C. Melanoma cells were selectively retrieved from tumor samples with PALM microlaser technology (PALM, Bernried, Germany) under microscopic control.

### Measurement of migration, attachment, and proliferation

The xCELLigence System (Roche, Mannheim, Germany) is based on measurement of electrical impedance and permits real-time analysis of migration, attachment, and proliferation. CIM plates (migration) and E-plates (attachment and proliferation) were used and basic protocols recommended by the manufacturer were followed. The bottom chambers contained culture supernatant from human fibroblasts as chemo-attractant. Upper chambers contained serum-free DMEM. After recording background impedance, cells suspended in serum-free DMEM were added to the upper chambers (4 × 10^3^/well for migration; 2 × 10^2^/well for proliferation/attachment). Thereafter, impedance can be measured continuously over 72 h or longer. Impedance is represented by the relative and dimensionless parameter named cell index (CI). CI values = Zi-Z0/15[Ohm]; where Z0 = impedance at the start of the experiment, and Zi = impedance at individual time points during the experiment. The normalized cell index (NCI) was calculated as the cell index CI_ti_ at a given time point (ti) divided by the cell index CI_nml_time_ at the normalization time point (nml_time). The slope is used to describe the steepness of a curve within a given time window (in our case 1.5h (attachment) 4 h (migration, and 72 h (proliferation)).

### Clonogenic Assay (stem cell behavior)

The *in vitro* cell survival assay based on the ability of a single cell to grow into a colony was performed like it was described by Franken et al. [38].

### Colony formation in soft agar (attachment independent growth)

For the measurement of attachment independent growth 2 × 10^4^or 4 × 10^4^ cells per well were seeded into 6 well plates in DMEM/10 % FCS in 0.3 % agar on top of a 0.5 % agar bed prepared in water. The cultures were incubated for 10 to 20 days at 37°C, 8 % CO_2_. After the chosen incubation time the number and the size of colonies was recorded under an inverted microscope.

### Tube formation (vasculogenic mimicry)

Growth factor reduced Matrigel (BD Biosciences) was added to eight-chamber polystyrene vessel tissue culture-treated glass slides (BD Bioscience, Heidelberg, Germany) and allowed to gelatinize for 20 min at 37°C. To assay vasculogenic mimicry 7 × 10^4^ melanoma cells were seeded onto Matrigel-coated culture slides. Tube formation was assessed by phase contrast microscopy after 16 h and recorded with a digital camera.

### Hypoxia

Membrane permeable Desferrioxamine (DFX) and 2, 2′-dipyridyl (DP) as iron chelators and inhibitors of Prolylhydroxylases (PHDs) were used in a concentration of 250 μM for DFX and 50 μM for DP diluted in DMEM.

### NRN1 ELISA

The human Neuritin ELISA kit for the quantitative determination of NRN1 in serum and tissue homogenates was purchased from CUSABIO Life Science (Hubei, China) and distributed from Hoelzel diagnostic (Cologne, Germany). The assay was performed like in the manufacture's instruction described. The serum collection for this study was approved by the medical ethical committee of the University of Erlangen-Nuremberg, and as prescribed patient identity and data privacy was protected. Furthermore, we used several sera from the study performed by Raja et al. 2007 [39].

### PRISURV

We used a free online datamining tool (http://www.bioprofiling.de/PPISURV). PPISURV automatically correlates expression of an input gene interactome with survival rates on > 40 publicly available clinical expression data sets covering various tumors involving about 8000 patients in total.

### Statistical analysis

All experiments were performed on at least 3 independent occasions. Results are given as mean ± SD. Unless stated in the figure legend comparison between groups was made using the Student's paired t-test. All calculations were performed using the GraphPad Prism software (GraphPad software Inc, San Diego, USA).

## SUPPLEMENTARY FIGURES


