# Supplementary material for: The neurotrophin Neuritin1 (cpg15) is involved in melanoma migration, attachment independent growth, and vascular mimicry
Source: Oncotarget. 2016 Nov 25;8(1):1117–31. doi: 10.18632/oncotarget.13585 (PMC5352040; doi:10.18632/oncotarget.13585)
Supplement: Supplementary file 1 [file oncotarget-08-1117-s001.pdf]

The neurotrophin Neuritin1 (cpg15) is involved in melanoma migration, attachment independent growth, and vascular mimicry

SUPPLEMENTARY FIGURES

A

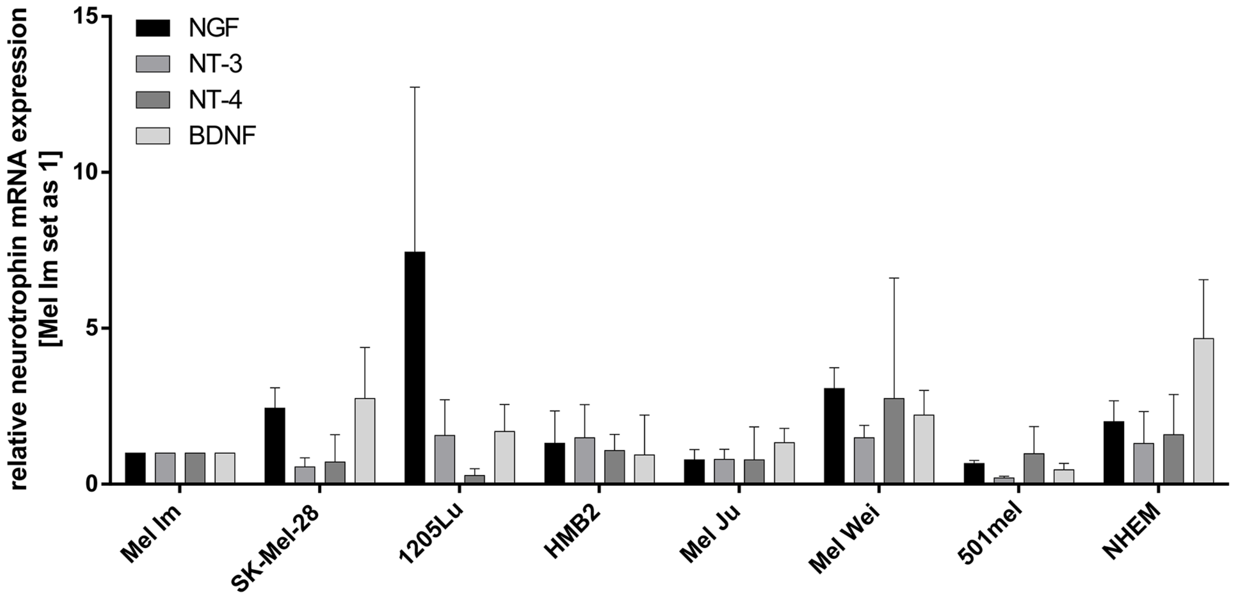

B

	NHEM	PT	MET
NGF	1	1,64	1,71
NT-3	1	1,44	1,28
NT-4	1	1,78	1,59
BDNF	1	2,08	1,27
NRN1-L	1	2,25	2,00
NRN1	1	57,9	38,2

Supplementary Figure S1: Expression level of neurotrophins. A. Expression analysis of the neurotrophins NGF, NT-3, NT-4, and BDNF in melanoma cell lines compared to NHEM. Data is mean +/- from triplicate experiments. **B.** cDNA array analysis of the neurotrophins NGF, NT-3, NT-4, BDNF, and NRN1-like in six melanoma cell lines compared to NHEM (relative units).

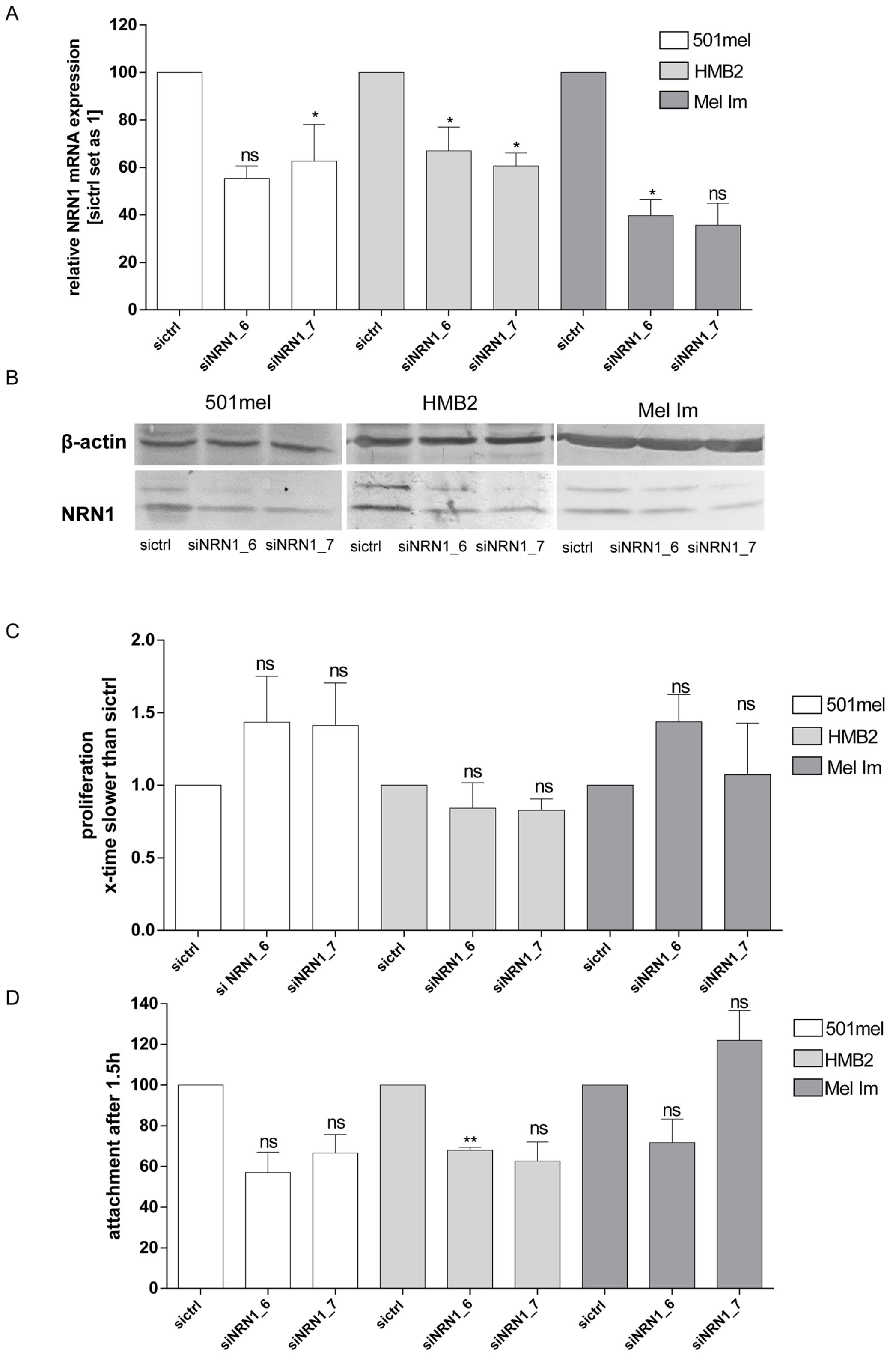

Supplementary Figure S2: Examination of NRN1 after siRNA application. A. and **B.** qRT-PCR and Western blot analysis for the expression level of NRN1 after usage of two specific siRNA (siNRN1_6, siNRN1_7) against NRN1. The knock-down was shown for three melanoma cell lines. The results are standardized to housekeeper gene β-actin in the qRT-PCR and it is the loading control of the Western blots. (ns, not significant; *p:<0.05). **C** and **D.** Proliferation and attachment of 501mel, HMB2, and Mel Im was analyzed with the xCELLigence system after usage of siRNAs against NRN1. (ns, not significant; **p:<0.01).

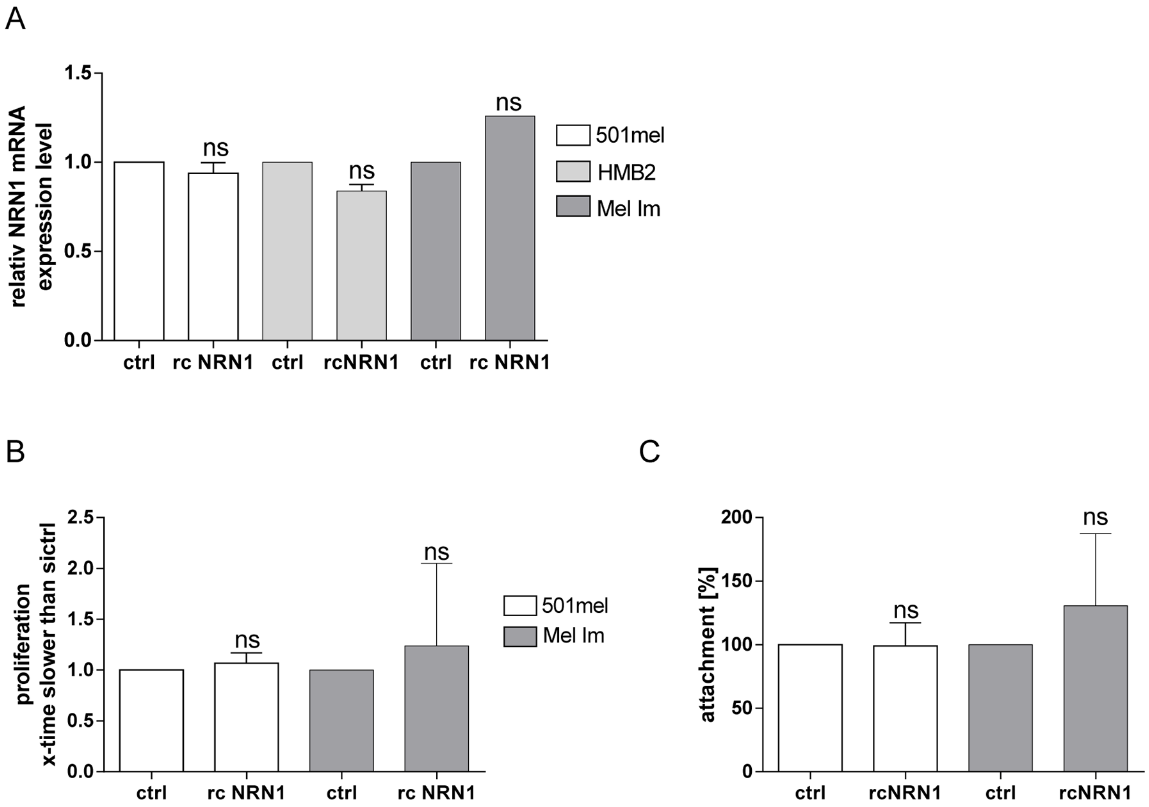

Supplementary Figure S3: Examination of NRN1 after application of recombinant NRN1. A. Quantitative real time PCR for endogenous NRN1 expression in melanoma cell lines 501mel, HMB2, and Mel Im after treatment of the cells with recombinant (rc) NRN1. (ns, not significant). **B.** and **C.** Proliferation and attachment of 501mel, HMB2, and Mel Im was analyzed with the xCELLigence system after treatment of the cells with recombinant (rc) NRN1. (ns, not significant).

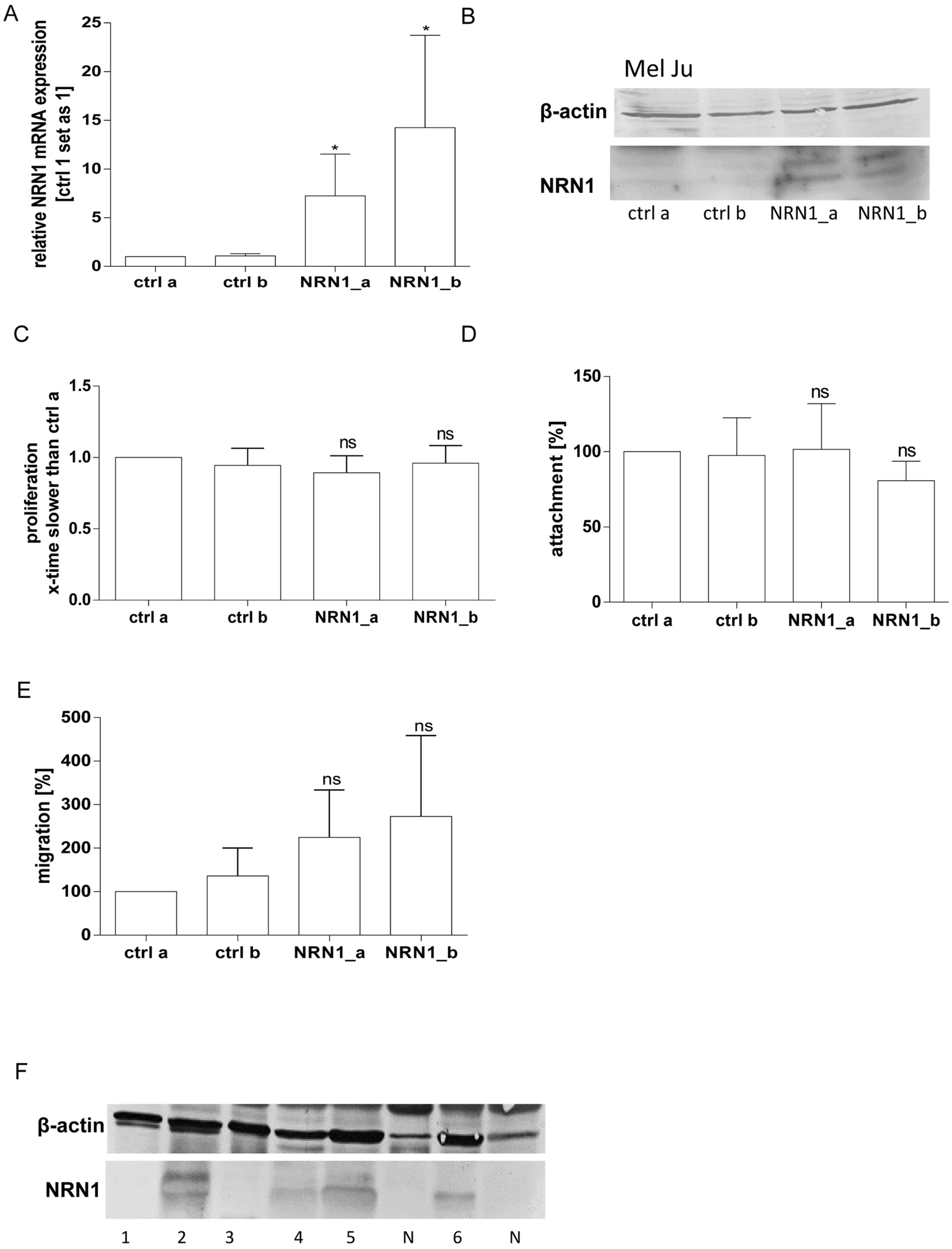

Supplementary Figure S4: Examination of NRN1 after overexpression of NRN1. A. and **B.** qRT-PCR and Western blot analysis detecting the expression level of NRN1 after usage of two different Mel Ju cell clones with stable NRN1 overexpression (NRN1_a, NRN1_b) in comparison to pCDNA3 control cell clones (ctrl a, ctrl b). The results are standardized to housekeeper gene β-actin in the qRT-PCR and it is the loading control of the Western blots. (*p:<0.05). **C.** and **D.** Proliferation and attachment of Mel Ju cell clones (ctrl a, ctrl b, NRN1_a, NRN1_b) were analyzed with the xCELLigence. (ns, not significant). **E.** xCELLigence system for analyzing the migration of Mel Ju cell clones (ctrl a, ctrl b, NRN1_a, NRN1_b) were analyzed with the xCELLigence. (ns, not significant). **F.** Western blot analysis of NRN1 in *in vivo* melanoma patient material (1-6) compared to normal skin (N).

A

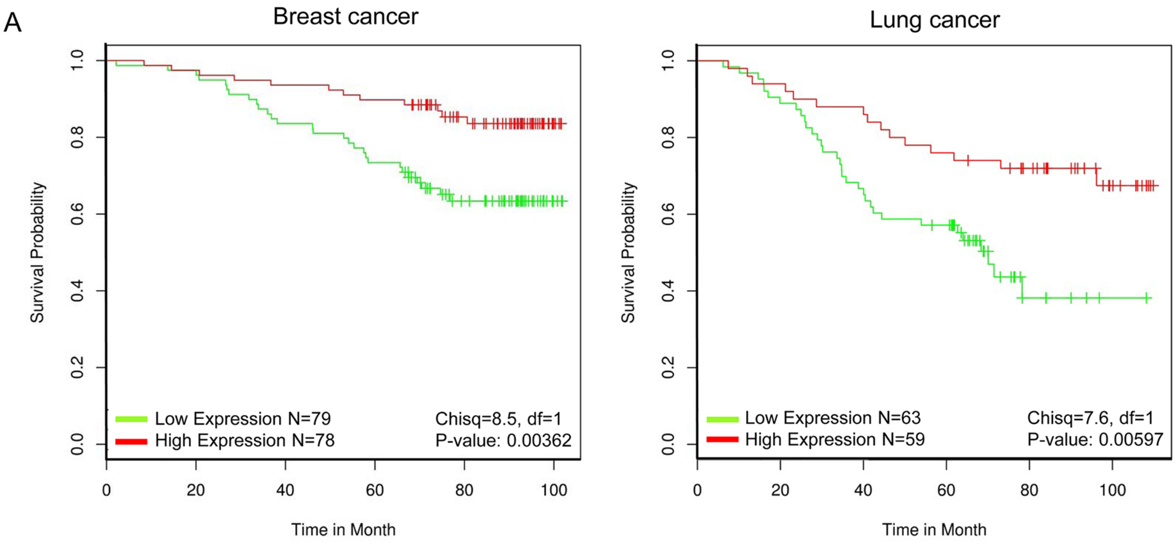

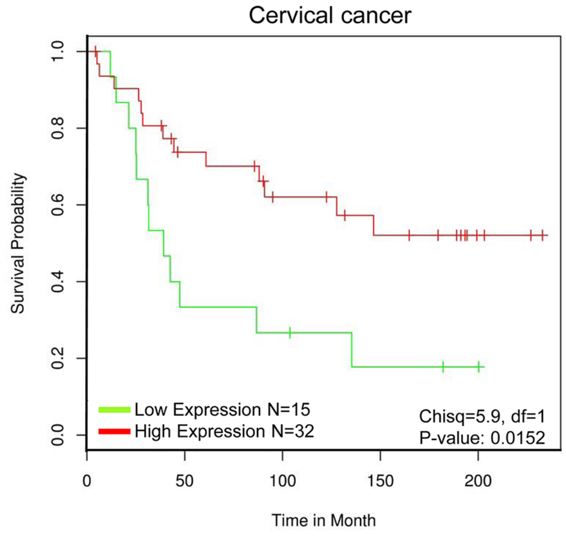

GEO dataset (GSE)	Cancer Type	GENE (probe ID)	P-value	Effect on survival
1456<br>2990<br>19783<br>17705	breast	218625_AT<br>218625_AT<br>A_23_P41451<br>9<br>218625_AT	0.003<br>0.009<br>0.053<br>0.055	Positive<br>Positive<br>Positive<br>Positive
13213	lung	A_23_P82088	0.005	Positive
30760	cervical	CG11504897	0.015	Positive

Criteria: P-value < 0.055; Gene NRN1

Supplementary Figure S5: NRN1 survival data of breast-, lung-, and cervical CA. A. Kaplan-Meier curves depicting disease specific survival of breast cancer, lung cancer, and cervical cancer patients with high (red) compared to low (green) expression (using multiple clinical datasets with PRISURV). Chisq= chi square, df= degrees of freedom.
